# Supplementary material for: A fault-intrusion-tolerant system and deadline-aware algorithm for scheduling scientific workflow in the cloud
Source: PeerJ Comput Sci. 2021 Nov 2;7:e747. doi: 10.7717/peerj-cs.747 (PMC8576554; doi:10.7717/peerj-cs.747)
Supplement: Supplemental Information 6 [file peerj-cs-07-747-s006.zip › Myproject1.1/docs/org/cloudbus/cloudsim/network/datacenter/class-use/NetworkCloudlet.html]

Uses of Class org.cloudbus.cloudsim.network.datacenter.NetworkCloudlet


JavaScript is disabled on your browser.


- Overview
- Package
- Class
- Use
- Tree
- Deprecated
- Index
- Help

- Prev
- Next

- Frames
- No Frames

- All Classes

## Uses of Class org.cloudbus.cloudsim.network.datacenter.NetworkCloudlet

- Packages that use NetworkCloudlet

  | Package | Description |
  | --- | --- |
  |  |  |
  | --- | --- |
  | org.cloudbus.cloudsim.network.datacenter |  |
- - ### Uses of NetworkCloudlet in org.cloudbus.cloudsim.network.datacenter

    Fields in org.cloudbus.cloudsim.network.datacenter with type parameters of type NetworkCloudlet

    | Modifier and Type | Field and Description |
    | --- | --- |
    |  |  |
    | --- | --- |
    | `java.util.ArrayList<NetworkCloudlet>` | AppCloudlet.`clist` |
    | `java.util.ArrayList<NetworkCloudlet>` | NetworkVm.`cloudletlist` |

    Methods in org.cloudbus.cloudsim.network.datacenter with type parameters of type NetworkCloudlet

    | Modifier and Type | Method and Description |
    | --- | --- |
    |  |  |
    | --- | --- |
    | `<T extends NetworkCloudlet>  java.util.List<T>` | NetDatacenterBroker.`getCloudletList()` Gets the cloudlet list. |
    | `protected <T extends NetworkCloudlet>  void` | NetDatacenterBroker.`setCloudletList(java.util.List<T> cloudletList)` Sets the cloudlet list. |

    Method parameters in org.cloudbus.cloudsim.network.datacenter with type arguments of type NetworkCloudlet

    | Modifier and Type | Method and Description |
    | --- | --- |
    |  |  |
    | --- | --- |
    | `void` | NetDatacenterBroker.`submitCloudletList(java.util.List<? extends NetworkCloudlet> list)` This method is used to send to the broker the list of cloudlets. |

- Overview
- Package
- Class
- Use
- Tree
- Deprecated
- Index
- Help

- Prev
- Next

- Frames
- No Frames

- All Classes
